# Cognitive Functioning in Adults with Phenylketonuria in a Cohort of Spanish Patients

**DOI:** 10.1155/2023/9681740

**Published:** 2023-02-08

**Authors:** P. M. Luna, J. F. López-Paz, M. García, I. Amayra, O. Martínez, M. Pérez, A. A. Rodríguez, P. Pérez-Núñez, I. Ceberio, N. Mansilla, C. Soria

**Affiliations:** ^1^University of Deusto, Department of Psychology, Av. de las Universidades 24, Bilbao 48007, Spain; ^2^Suportias, Nursing Department, Av. Juan Carlos I, 13, Madrid 28806, Spain; ^3^Suportias, Health Psychology Department, Av. Juan Carlos I, 13, Madrid 28806, Spain

## Abstract

The early introduction of a low phenylalanine (Phe) diet has been demonstrated to be the most successful treatment in subjects with phenylketonuria (PKU), especially for preventing severe cognitive and neurological damages. However, it still concerns that even if treated in the first months of life with supplements and following a diet, they can show slight scores below people without PKU in neuropsychological assignments. We investigated 20 adults with classical PKU aged 19–48 years (mean age 29 years) and 20 heathy controls matched by age, gender, and years of education. Patients and controls were assessed with an extended neuropsychological battery, as well as psychological aspects and quality of life, also the last Phe level result was obtained. Results showed that the most affected cognitive domains are processing speed, executive functioning, memory, and also theory of mind, but very well-preserved verbal fluency, language, and visuospatial functioning. In quality of life, some significant results were seen specially in anxiety of Phe levels, anxiety of Phe levels during pregnancy, guilt if poor adherence to supplements, and if dietary protein restriction not followed. No significant results were obtained for the psychological variables. In conclusion, it has been shown that a combination of a low Phe diet, supplement intake, and keeping Phe levels in a low range seems appropriate to have the most normal and alike cognitive performance to persons without PKU.

## 1. Introduction

Phenylketonuria (PKU) is the most frequent inborn metabolic error, with an incidence of 1 in 10,000 on average in Europe [1]. This disease occurs as the result of a disruption of the phenylalanine hydroxylase enzyme (PHA), which is responsible for metabolizing the amino acid phenylalanine (Phe) into tyrosine (Tyr) [2]. This loss in PHA activity causes blood Phe accumulation, toxic Phe concentrations in the brain, and Tyr deficiency [3].

Regarding the clinical manifestations of PKU patients, usually some developmental delay, severe cognitive and psychiatric impairments can arise if PKU is left untreated [4]. Some cognitive domains such as attention [5, 6] and psychomotor speed are usually affected even in early treated adults Aitkenhead et al., [49], as well as in other domains such as language, memory, learning, deficits in fine motor control, processing speed, and attention control [7]. Some deficits in working memory [5, 8] and executive functions such as problem-solving, planning ability, inhibitory control, and mental flexibility have been reported as well in adults after they stopped or relaxed the diet (Pascucci et al., 2009, 2013).

If left untreated during childhood, PKU can cause microcephaly, seizures, and behavioral problems [6]. They can also experience other types of problems such as depression, anxiety, poor self-image, and mood swings 5 [4, 9]. These emotional difficulties might reduce the person's ability to cope with the daily demands, creating more mental health alterations [10].

It is known that the most common treatments for PKU individuals are restrictive protein diet and supplements. There is evidence in literature such as the Trepp et al. [11] study who refer to ten Hoedt et al., [62] that showed that following a low Phe diet is essential since being diagnosed and throughout life to avoid cognitive deficits. Pascucci et al., (2012) refer that even having mildly elevated blood Phe levels might affect the cognitive functions, especially those related to the prefrontal cortical area, so if there does not exist a dietary modification [55], PKU can cause irreversible cognitive deficits and structural brain damage.

Having a low Phe diet and control over Phe levels for life is necessary to have a proper cognitive disengagement, according to Dawson et al. [12], where their data showed that adults with an unrestricted diet had significantly lower reaction times compared to the control group, and on the other hand, those who were in a protein restricted diet with 800 *μ*mol/l or less did not differ significantly in reaction times from the control group. Romani et al. [35] mentioned that fluctuations throughout life have a significant impact on some cognitive measures, which supports the importance of knowing this parameter, especially in the domain of sustained attention where the impact of Phe levels has been directly related, but not in learning and memory. This has been reflected not just in clinical studies but also in functional magnetic resonance imaging (fMRI), where Abgottspon et al. [13] studied the effect of Phe on cerebral markers in a group of individuals with PKU and found that the task accuracy was lower in patients with PKU, but that the reaction time between these patients and the control group was similar. Even though fMRI studies have been conducted in PKU population, Trepp et al., [11] mention that there is still a limited amount of studies with this technique, which creates uncertainty in AwPKU between the relationship with Phe concentrations and the brain abnormalities that can occur.

Reports related to psychosocial outcomes as mentioned by Aitkenhead et al., [49] suggest occupational functioning deficits as well as low academic attainment (Bosch, et al. [14]) in adults with PKU.

Mental health problems such as anxiety disorders, mood swings, and depression have been associated with elevated Phe levels [6], as well as poor self-image and overall behavioral problems [9].

Referring to quality of life (QoL), Alptekin et al., (2018) mention that living with this chronic illness can contribute to psychological and psychiatric symptoms in individuals, which affects directly their QoL, having as well problems in social sphere because of the diet rules, carrying supplements, and formulas.

According to the European guideline (Wegberg et al., [63]), the suggested level of Phe for AwPKUs should be maintained under <600 *μ*mol/l from >12 years old, being the main goal of treatment to achieve a normal function in neurocognitive and psychosocial areas. If levels are above >600 *μ*mol/l, treatment should be considered. This is why, considering the available research, it's clear that the most effective treatment is a restricted intake of protein for life, along with taking the supplements of the dietary formulas, which help reaching a balance of other amino acids, minerals, and vitamins that are essential for a correct development [4].

In this metabolic disease, there is usually a shortage of certain amino acids such as Tyr and tryptophan, for instance, there are lower concentrations of the neurotransmitter's serotonin and dopamine. Dopamine is important for the proper function of the executive functions [55] and if there is a dysfunction in this area, there may be problems related to planning and inhibitory control. As well as decreased levels of serotonin may be associated with other cognitive problems and mood disorders [9].

According to Pilotto et al. [15], during childhood and also in adulthood, it has been indicated that there is a significant relationship between having a poor control of Phe levels and disrupted neuropsychological abilities. During childhood, according to Romani et al. [3], if a Phe restricted diet and Phe free amino acid supplementations are followed since birth, these negative consequences can be controlled; however, around 70% of AwPKU do not attend to their regular clinical appointments [16] so that the adherence and relaxation of the diet decrease [4], even though Phe control for life is recommended, even during adulthood.

Adults with this metabolic disease, usually present slight results below healthy control groups in intelligence (Anderson et al., [17, 18, 50]), also in reduced processing speed and dysfunction in executive functioning tasks (Anderson et al., [18–20, 50]). Bartus et al. [4] also mention a reduced processing speed, difficulties with working memory, reduced IQ, and attention problems. Adults have presented deficits mostly in the verbal area, sustained attention, and reaction time [21, 55].

As it can be observed, more research needs to be developed to understand specifically how PKU affects the cognitive and psychosocial domain. This is why, the aim of this study is to investigate a cohort of AwPKUs in Spain, on their cognitive performance in comparison to age, sex, and years of education matched with healthy controls. Secondarily, the study aims to assess the emotional health, behavior, and QoL on these subjects.

## 2. Method

### 2.1. Participants

Twenty AwPKUs participants, 3 males and 17 females aged 19–48 years (*M* = 29.55, SD = 8.81) with classical PKU participated in the study. Sixteen were recruited through FEDER (Spanish Federation of Rare Diseases) and different associations of PKU in Spain (Avapku, Asociación de Familias con Enfermedades Metabólicas de Madrid. Association of Families with Metabolic Diseases of Madrid (ASFEMA)), and 4 participants were referred from hospitals (Hospital Clínico Universitario de Santiago, Hospital Universitario Miguel Servet) through the PKU Support Program. Their diagnosis was confirmed through newborn screening and have been treated with a low Phe diet and supplements since diagnosis. By the time of the testing, 16 were still following a low Phe diet, while 4 participants were not following a restricted diet. Even though lifetime Phe levels were not available for some, recent blood levels were obtained from all participants. All of this information was extracted from the report each patient handed.

AwPKUs participants were compared to a group of 20 healthy controls, 3 males and 17 females (*M*_age_ = 30, SD_age_ = 9.43), which were recruited via researchers, and matched by age, gender, and years of education.

Sample recruitment took place between July 2020 and September 2021. Sociodemographic and clinical data are shown in Table 1. There were no statistically significant differences between the two groups with respect to gender, *χ^2^*(1) = 0, *p* = 1; age, *U* = 195.0, *p* = 0.892; nor years of education, *U* = 138.5, *p* = 0.094.

The inclusion and exclusion criteria were the following: *Inclusion criteria:* (a) being over 18 years of age; (b) resident in Spain; (c) having fulfilled the informed consent document prior to the assessment; and (d) having a diagnosis of PKU confirmed by a doctor specialized in metabolic diseases. *Exclusion criteria:* (a) intellectual disability; (b) having any another neurological, psychological, or psychiatric diagnosis not secondary to PKU; (c) not giving their consent to participate in the study. Regarding the control group, the inclusion and exclusion criteria were the same, except for the diagnosis of PKU.

### 2.2. Procedure

Participants who were interested in participating in this study (recruited through FEDER and the Associations) were informed about the process of evaluation via email or call, with a dossier that included information about the research group, specifying the objectives of the study, duration of the neuropsychological evaluation, and how to participate. Participants referred from the PKU Support Program had to sign the specific informed consent of the Program. They were tested via online in one session, lasting approximately 1 hour 45 minutes each. Both the clinical and the control group were administered the same tests, except for the PKU QoL. The clinical information of the patients, such as Phe levels, was collected from the clinical reports given by the individuals and referred to their last evaluation or last control, relative to the last 6 months. The participation of all members of the sample was voluntarily, signing an informed consent to take part in the research that was approved by the University of Deusto Ethics committee (ETK-33/18-19) and also by the Organic Law 15/99 of the 13^th^ December of Spanish Law regarding the Protection of Personal Data and the Declaration of Helsinki (Edinburgh, 2000).

### 2.3. Data Analysis

The statistical analyses were carried out using IBM SPSS Statistics 28.0 software. *P* value less than 0.05 was considered as statistically significant. Raw scores were converted into *z* score to run the analyses. To verify the homogeneity of the groups, Chi-square test was used for gender and *U* Mann–Whitney for age and years of education. To analyze sociodemographic and cognitive variables between both the clinical and control, *U* Mann–Whitney was used. Pearson's correlation coefficient (*r*) was used to calculate the effect size. A multiple regression analysis was used to analyze the influence of variables related to the PKU diagnosis on cognitive performance.

## 3. Instruments

### 3.1. Cognitive Functioning

Participants completed a large battery of neuropsychological tests, and the following cognitive domains were evaluated. Executive functions: (a) backward digit subtest from the Wechsler Adult Intelligence Scale-IV from the WAIS-IV (Spanish version—[22, 23]); (b) *Stroop Color-Word Test* ([24]; Spanish version—[25]). Verbal fluency: (a) phonetic verbal fluency from the *Controlled Oral Word Association Test—COWAT* (F-A-S; [26, 61]); (b) semantic verbal fluency test from the *COWAT* (“Animals”; [26, 61]). Visuospatial functioning: (a) matrix reasoning subtest from the WAIS-IV (Spanish version—[22, 23]); (b) incomplete figures subtest from the WAIS-IV ([22]; Spanish version—[23]); (c) puzzles subtest from the WAIS-IV (Spanish version—[22, 23]); (d) copy accuracy and copy time from the *Rey–Osterrieth Complex Figure* Test (ROCF; [27]; Spanish version—[28]). Language: (a) similarities, vocabulary, information, and comprehension subtests from the WAIS-IV ([22]; Spanish version—[23]). Memory: (a) direct digit subtest from the WAIS-IV ([22]; Spanish version—[23]); (b) visual memory from the ROCF([27]; Spanish version—[28]). Processing speed: *Symbol Digit Modalities Test* (SDMT; Smith, 1968; Spanish version—[29]). Theory of mind: *Happé's Strange Stories Test* ([30]; adapted by [31]).

### 3.2. Emotional Health, Behavior, and Quality of Life

Participants completed the following questionnaires. Depression: *Beck Depression Inventory-II* (BDI-II; Beck, Steer, & Brown [52]; Spanish version—Sanz, [59]). Quality of life: *Adult PKU-QoL Questionnaire* (Regnault [32]). Behavioral and emotional disturbances: *Adult Self Report 18-59* (ASR; [33]).

## 4. Results

As shown in Table 2, there were differences statistically significant between AwPKUs and healthy controls in the following tasks: executive functioning, specifically in the Stroop test, in color word, and interference; perceptual reasoning, in the copy accuracy (ROCF); memory, in the subtest of digits direct, as well as in visual memory (ROCF); processing speed task (SDMT); and in theory of mind (ToM; Happé's Strange Stories Test).

In the psychological area, the results in the BDI-II did not show any differences statistically significant (*U* = 157.5, *p* = 0.250). Secondly, no significant differences were shown ASR (*U* = 181.0, *p* = 0.607).

The differences between AwPKUs with levels above (*n* = 11) and below (*n* = 9) 600 *μ*mol/l were analyzed. The results between the cognitive and psychological aspects in this two groups were statistically significant in the next subtests of the WAIS-IV: similarities (*U* = 20.0, *p* = 0.024), matrix (*U* = 23.5, *p* = .047), vocabulary (*U* = 11.5, *p* = 0.004), information (*U* = 10.5, *p* = 0.003), comprehension (*U* = 20.0, *p* = .024), and incomplete figures (*U* = 14.5, *p* = 0.007). As well as in the SDMT Total (*U* = 19.0, *p* = 0.020) and FAS (A) (*U* = 21.0, *p* = 0.029).

According to the results obtained in the PKU QoL (clinical group), some of the variables showed results above 50 (the higher the result, the lower QoL in that domain). In the symptoms area, the self-rated health status (*M* = 57.50, SD = 30.45). In relation with the PKU in general, the anxiety Phe levels (*M* = 63.75, SD = 29.77), anxiety Phe levels during pregnancy (*M* = 55, SD = 44.12), and PKU information (*M* = 51.25, SD = 34.86). Related to the supplement administration, guilt if poor adherence (*M* = 59.50, SD = 35.94) and taste of the supplements (*M* = 53.25, SD = 32.85). Finally, in the dietary protein restriction area, guilt if dietary protein restriction is not followed (*M* = 60, SD = 31.83) and food enjoyment (*M* = 85, SD = 20.51).

Multiple regression analysis was carried out to assess the influence of clinical variables on the cognitive performance of the clinical group. The impact of diagnosis age (<1 month, 1–12 months, 12–24 months, >2 years), diet, and the intake of supplements were included, as well as the variables of the PKU QoL questionnaire above 50.

As the clinical group has demonstrated a statistically significant deficit higher than the healthy control group, we wanted to analyze the influence of certain clinical variables likely to affect their cognitive performance. For this, the following multiple regression analysis has been applied. The model has been fulfilled for total digits (*F* = 13.17, *R*^2^ = 0.958, *p* ≤ 0.001); however, the model has not been fulfilled for ROCF copy (*F* = 2.29, *R*^2^ = 0.797, *p* = 0.138); ROCF memory (*F* = 1.30, *R*^2^ = 0.690, *p* = 0.376); Stroop Color-Word (*F* = 1.59, *R*^2^ = 0.733, *p* = 0.273); SDMT (*F* = 3.21, *R*^2^ = 0.846, *p* = 0.065); Happé's Strange Stories Test (*F* = 1.52, *R*^2^ = 0.723, *p* = 0.295). The significant predictors included in the model were: in the last 7 days followed PKU diet (*β* = 0.991, *p* ≤ 0.001), in the last 7 days stopped taking supplements (*β* = −0.365, *p* = 0.032), stopped taking supplements because of working limitations (*β* = −0.487, *p* = 0.043), Phe levels (*β* = −0.576, *p* = 0.004), diagnosis age (*β* = −0.896, *p* ≤ 0.001), following a diet (*β* = −1.155, *p* = 0.002), and supplements intake (*β* = 0.716, *p* = 0.004).

As seen, the model was not met for Stroop test, ROCF, SDMT, and Happé's Strange Stories Test, so that the performance of the group on executive functioning, visuospatial abilities, visual memory, processing speed, and ToM tasks was independent of the clinical impact.

## 5. Discussion

Even though there has been plenty of research related to PKU in adults [34, 55], still more studies are needed. Frequently, adults who started treatment right after birth have demonstrated better performance in cognitive tasks, being usually in the normal range and capable of living independently. This is the opposite for some adults with PKU, who have been diagnosed late or does not follow a strict diet, therefore have neuropsychological deficiencies, affecting their QoL [8]. Another important thing that can impact in the life of this subjects, according to Romani et al. [3], is the importance of keeping low Phe levels (<600 *μ*mol/l), so that AwPKUs cognitive performance can be nearly as good as controls.

Elevated levels of Phe have confirmed to cause brain toxicity, which can result in cognitive deficits. In the present study, the neuropsychological performance of a group of 20 AwPKUs was compared to 20 healthy controls, matched by gender, age, and years of education. We also looked at whether there were any differences between the subgroups of patients in the clinical group, according to the levels of Phe. Considering the entire neuropsychological assessment, the AwPKUs performed well in some of the cognitive domains, but there were some deficits in some others, such as executive functioning, visuospatial functioning, memory, processing speed, and ToM.

### 5.1. Cognitive Deficits

The results in the present study show deficit in the memory domain (digits direct of WAIS-V, visual memory of the ROCF) in the AwPKUs, which matches the results in Ashe et al., [8] and Channon et al. [53] studies. Bartus et al. [4] and Pilotto et al. [15] assessed memory tasks to AwPKU population, obtaining as well, lower scores compared to healthy controls, while Bilder et al. [16] and Palermo et al. [35] studies did not show any differences between the clinical and control group in this domain.

According to the literature available, the processing speed domain has been consistently the primary deficit in the PKU adult population [8]. The reason for this, according to Moyle et al. [55], is because of the role Phe concentration and elevated plasma levels play in the brain that affects the process of myelination. The result obtained in the present study was that the clinical group had lower scores in this domain compared to the healthy controls, just like in Janos et al. [36] and Palermo et al. [10] studies. But, this domain is not altered in the study of Bartus et al. [4]. Romani et al. [37] make an interesting observation about this domain, where their study results do not show a generalized processing speed reduction in AwPKUs, arguing that the neural metabolism disruption that occurs in PKU may affect the speed of processing in the different cognitive domains, but not just processing speed per se, just like in Hofman et al. [5] study, concluding that the fastest AwPKUs were just as fast as the fastest healthy controls. Also, Palermo et al. [35] indicate that the slowing in processing speed occurs specially at the cognitive levels when there is a toxic high level of Phe on the oligodendroglia in the central nervous system. Dawson et al. [12] mentioned that they found in studies that in adults the results show that the reaction times were not so different from people without PKU, this was despite they were or not on a low Phe diet restriction, but in their own study, the results were that AwPKUs in off-diet had a slower processing speed than controls. The difference between the results in the present study and the results obtained in Bartus et al. [4] and Romani et al. [37] might vary depending on the Phe levels of the AwPKUs at the moment of the evaluation.

Impairments in the executive functions affect the everyday life basic cognitive tasks like planning, being able to focus and to have impulsive control and may also hide the efforts of following a restricted diet, monitor Phe levels, plan, and schedule [16]. The results in this study showed that AwPKUs had a lower performance specifically in color-word (Stroop), which is the same result as in Bik-Multanowski et al. [37] because they did find that inhibitory control in patients with PKU was less effective. In digit backward subtest from the WAIS-IV, we did not find any deficits just like in Anderson et al. [38] and Felice et al. [1] who did not find deficits in this area as well, where they find an intact performance in children with PKU treated in the first months of life in this task. Other studies [10] did find impairments in the executive function domain, specifically in tasks involving planning and monitoring.

Finally, the ToM domain did showed deficits just like in Ashe et al. [8] and Jahja et al. (2016) who also reported difficulties in this area, arguing that it can probably be linked to elevated Phe levels.

### 5.2. Not Affected Cognitive Abilities

The results obtained in verbal fluency showed no significant differences, which is consistent with Moyle et al. [55] and Palermo et al. [35] results. But the opposite result was obtained by Brumm et al. [21], where verbal fluency results were shown as a weakness for AwPKUs. The same results were obtained in visuospatial function, which are the opposite compared to Pennington et al. [39], Palermo et al. [35], and Palermo et al. [10], where their results presented significant lower scores.

The results obtained in language domain were evaluated with similarities, vocabulary, information, and comprehension subtests from the WAIS-IV and showed as well no differences statistically significant between the AwPKUs and the healthy control group. These results are the same as in Palermo et al. [35] and Felice et al. [1], where no impairments were found and basic language skills had normal performance, but deficits were shown in the AwPKUs complex language tasks performed. Meanwhile, Brumm et al. [21] found impairments in vocabulary and similarities. Felice et al. [1] mentioned the lack of study in adult population in the language domain between language performance and Phe levels, where there has been shown inconsistency in the results. In child population (see ref. [40]), there are studies only with late treated but not treated in the first's months of life.

The possible explanation to these results is maybe because of the lack of other tests applied in this area, having only one, Happé's Strange Stories and the small sample might not be enough.

### 5.3. Subgroup Analysis

As referred before, the new European guidelines target the ideal blood Phe levels under 600umol/l to have a good cognitive function. This is why we decided to add an additional result, dividing the patients in subgroups depending on their last Phe levels result. In this study, 9 patients had Phe levels above >600 *μ*mol/l and 11 under <600 *μ*mol/l. A few participants had Phe levels above the required, but demonstrated to have a good cognition. This was also shown in Romani et al. [3], where some individuals had poor metabolic control in adulthood, but demonstrated good cognition results, supporting the idea that some individuals might have some protective factors where high Phe levels does not affect them. Hofman et al. [5] stated that AwPKUs treated in the first months of life with concurrent low Phe levels performed better than AwPKUs treated in the first months of life with concurrent high Phe levels, especially on tasks like memory and language skills. In the present study, the results showed lower scores in participants with high Phe levels >600 *μ*mol/l in language (similarities, vocabulary, comprehension, information from the WAIS-IV) but not in memory, as well as deficits in perceptual reasoning (matrix and incomplete figures from the WAIS-IV), processing speed (SDMT), and verbal fluency (FAS).

### 5.4. Psychological and Behavioral Symptoms

Because of Choi and Pardridge [41], we know that the disruption suffered in PHA damages the production of Tyr coming from Phe. This amino acid shares the same transport system with another amino acids (Tyr and tryptophan) to get to the blood–brain barrier, so that Phe limits the entry of these in the central nervous system, causing a critical impact and dysregulation in depressive symptoms, anxiety, overall mood, and cognition ([54] [42]).

As seen in van Spronsen et al. [43], deficiencies caused in the neurotransmitters monoamine and norepinephrine were suggested as pathophysiological mechanisms that might cause deficits in the behavioral and neuropsychiatric symptoms. A very common psychological symptom of AwPKUs appears to be depression, even in adults treated in the first months of life [15]. Bueno et al. [44] obtained similar results arguing that half of their late diagnosed patients presented depression, as well as Bilder et al. (2017) who demonstrated that depression symptoms are higher in PKU population compared to people without PKU.

In one hand, Ashe et al. [8] mention that the most common neuropsychiatric symptoms (depressed mood, social isolation, generalized anxiety, phobias, social maturity deficits, lower positive emotions, low self-esteem, and lack of autonomy) correlate with Phe levels. This psychological disability could be attributed not only to elevated blood Phe levels, but also to what living with a chronic disease can cause to the QoL to the person with PKU.

In the present study, no correlations or significant scores were obtained related to the BDI-II, being these results consistent with Brumm et al. [21] and Palermo et al. [10], who showed the same data where no clinically significant results for depression (BDI-II) were present and no relation between Phe levels and emotional health was found. Burgess et al. [19] findings suggest that these psychiatric deficits can be positive modified in AwPKUs, and that they are possible if the dietary control is followed up or improved.

To measure behavioral problems in AwPKUs, the Achenbach System of Empirically Based Assessment (ASEBA) for adults (ASR) was used. Jahja et al. [9] found in their study that AwPKUs presented internalizing problems in different from controls, even if they were following a strict treatment since childhood. According to Vardy et al. [64], recent studies highlighted a low self-esteem, withdrawal, and signs of anxiety in PKU population. Despite these results in different studies, the results obtained did now show any deficiency in the dimensions of the ASR or even in the global punctuation. As seen in Brumm et al. [45], patients with PKU who were untreated presented aggressive disorders, as well as autism and behavioral problems.

### 5.5. Quality of Life

It is known that having a chronic disease is usually associated to a lower QoL, and that if an AwPKU has a poor metabolic control, there will be higher psychological impairments. According to Thiele et al. [46] and Alptekin et al. [47], if this occurs, a poorer adherence to diet and supplements is more likely to happen, which supports our results in the PKU QoL questionnaire having obtained deficits in supplement administration, guilt if poor adherence to supplements, as well as guilt if dietary protein restriction is not followed. Our results match as well with Bosch et al. [48] where they mention that in all ages, the highest scores are in domains related to emotional impact of PKU, the management, guilt if poor adherence to diet, and supplement intake.

Another variable that has importance in the QoL of these individuals, and which is shown to be a strong symptom in AwPKUs compared to healthy controls, is anxiety. It can be explained by the following reasons: high blood Phe levels, low serotonin levels in the brain, and anxiety as a psychological stressor to follow up a strict diet and having adherence to it (Bilder et al., [52]).

This might support the results obtained regarding information, anxiety if having high Phe blood levels, and the blood Phe levels during pregnancy (considering that 17 patients from our study were woman) in the PKU QoL questionnaire, as also seen in Alptekin et al. [47] where the highest score for adolescents and adults was anxiety blood test, blood Phe levels, and information. Despite having obtained these results, the overall scores in the QoL questionnaire scored within the normal range without significant differences from healthy controls.

## 6. Limitations and Perspectives

The main limitation in our study is the low number of participants, because of PKU being a rare disease it is difficult to recruit a large sample, as well as being able to have access to a long-term blood biomarkers data of the clinical group for this analysis and not just the last Phe levels obtained. MRI studies would have also been interesting to add to this study and to be able to correlate them with Phe levels and cognitive results.

Another limitation is that this is a cross-sectional study, and it could be interesting doing a longitudinal research to see if the cognitive functioning in the long term, varies. Moreover, the dates when the study was carried out coincided with the COVID-19 pandemic, which could have impacted on the psychosocial state of patients and controls. Likewise, the lack of homogeneity in the sample is due to the fact that the individuals who were more interested in participating were females, which leads to not having an equivalent gender distribution. Finally, more research and studies in adult PKU population in Spain could be interesting, as well as doing international collaborations to access bigger samples. These limitations are considered to be addressed in further research.

## 7. Conclusions

We carried out a neuropsychological assessment of a sample of adults with PKU, using a variety of tasks across different domains. We have found that the clinical group obtained lower scores in some domains, including inhibitory control, visuospatial functioning, processing speed, and ToM. To our knowledge, studies in PKU have made clear that being early treated and keeping Phe levels in a low range seem appropriate to have the most normal and alike cognitive performance to persons without PKU. Our results highlight the need to examine more the adult PKU population in the neuropsychological area in bigger studies, including a larger number of participants to see if the deficits are also impacted because of brain ageing. In addition, more research in QoL in adults should be done, maybe that could increase patients' interest in keeping the diet for long term.

## Figures and Tables

**Table 1:** Distribution of the sample according to sociodemographic and clinical characteristics.

	Clinical group (*n* = 20) *M* (SD)	Control group (*n* = 20) *M* (SD)
Sociodemographic data		
Gender	17 females (85%)	17 females (85%)
	3 males (15%)	3 males (15%)
Age	29.55 (8.80)	30 (9.42)
Years of education	15.25 (3.71)	16.80 (3.25)
Clinical data		
Phe levels^a^ (*n* = 20)	585.75 (419.73)	
<600 *μ*mol/l (*n* = 11)	375.45 (125.39)	
>600 *μ*mol/l (*n* = 9)	991.67 (312.47)	
Diagnosis age^b^		
<1 month	15 (37.5%)	
1–12 months	2 (5%)	
12–24 months	1 (2.5%)	
>2 years	2 (5%)	

Note. ^a^Phe levels refer to the last blood Phe concentrations.

^b^Diagnosis age refers to the age they were when they received the Phenylketonuria diagnosis confirmation.

**Table 2:** Clinical and control group performance on cognitive tasks.

	Clinical group		Control group		*Z* scores		Effect size
	*n*	*M* (SD)	*n*	M (SD)	*U*	*p*	*r*
Executive functioning							
Digit backward	20	7.85 (2.35)	20	9.15 (1.95)	−1.537	0.124	0.28
Stroop (CW)	20	43.10 (11.11)	20	52 (11.89)	−2−260	0.024^b^	0.36
Interference	20	5.01 (10.80)	20	11.28 (8.80)	−1.92	0.055	0.30
Verbal fluency							
FAS	20	38.65 (12.81)	20	45.10 (7.99)	−1.665	0.096	0.28
Animals	20	23.10 (6.14)	20	26.25 (3.74)	−1.779	0.075	0.29
Visuospatial function							
Matrix	20	17.40 (5.82)	20	18.35 (4.50)	−0.339	0.734	0.09
Incomplete figures	20	10.60 (4.90)	20	12.85 (3.99)	−1.616	0.106	0.24
Puzzles	20	15.45 (6.65)	20	17.75 (4.65)	−1.14	0.254	0.19
ROCF							
Copy accuracy	20	29.50 (4.74)	20	34.20 (1.88)	−3.583	0.001^b^	0.54
Copy time	20	3.35 (1.77)	20	2.77 (1.64)	−0.907	0.364	−0.16^a^
Language							
Similarities	20	20.75 (5.23)	20	23.95 (5.72)	−1.939	0.052	0.28
Vocabulary	20	33.65 (10.63)	20	37.80 (7.55)	−0.880	0.379	0.21
Information	20	10.85 (5.82)	20	12.25 (4.54)	−1.071	0.284	0.13
Comprehension	20	22.50 (5.54)	20	24.95 (4.38)	−1.521	0.128	0.23
Memory							
Digits direct ROCF	20	7.60 (2.37)	20	10.30 (2.64)	−2.970	0.003^a^	0.47
Visual memory (immediate recall−3 minutes)	20	21.20 (7.13)	22	25.85 (4.61)	−2.236	0.025^a^	0.36
Processing speed							
SDMT	20	50.30 (12.58)	20	59.80 (8.57)	−2.627	0.009^a^	0.40
Theory of mind							
Happé's Strange Stories	20	11.40 (2.58)	20	13.40 (1.54)	−2.519	0.012^a^	0.42

M: Mean; SD: Standard Deviation; *U: U* Mann–Whitney; CW: Stroop Color-Word; ROCF: Rey–Osterrieth Complex Figure Test; SDMT: Symbol Digit Modalities Test. Note: Raw scores are shown.

^a^ *P* < 0.05.

^b^ *P* < 0.001.

## Data Availability

The data that support the findings of this study are available from the corresponding author [Luna, P.M.], upon reasonable request.
